# Glucose-Insulin-Potassium Alleviates Intestinal Mucosal Barrier Injuries Involving Decreased Expression of Uncoupling Protein 2 and NLR Family-Pyrin Domain-Containing 3 Inflammasome in Polymicrobial Sepsis

**DOI:** 10.1155/2017/4702067

**Published:** 2017-03-27

**Authors:** Jun-liang Zhang, Yi-ting Chen, Guang-dao Chen, Tao Wang, Ju-xin Zhang, Qi-yi Zeng

**Affiliations:** ^1^Center of Pediatrics, Zhujiang Hospital, Southern Medical University, Guangzhou, Guangdong 510280, China; ^2^Department of Neonatology, Nanfang Hospital, Southern Medical University, Guangzhou, Guangdong 510515, China; ^3^Clinical Laboratory Center, Nanfang Hospital, Southern Medical University, Guangzhou, Guangdong 510515, China; ^4^Department of Pediatrics, Central Hospital of Panyu District, Guangzhou, Guangdong 511486, China

## Abstract

Uncoupling protein 2 (UCP2) may be critical for intestinal barrier function which may play a key role in the development of sepsis, and insulin has been reported to have anti-inflammatory effects. Male Sprague-Dawley rats were randomly allocated into five groups: control group, cecal ligation and puncture (CLP) group, sham surgery group, CLP plus glucose-insulin-potassium (GIK) group, and CLP plus glucose and potassium (GK) group. Ileum tissues were collected at 24 h after surgery. Histological and cytokine analyses, intestinal permeability tests, and western blots of intestinal epithelial tight junction component proteins and UCP2 were performed. Compared with CLP group, the CLP + GIK group had milder histological damage, lower levels of cytokines in the serum and ileum tissue samples, and lower UCP2 expression, whereas the CLP + GK group had no such effects. Moreover, the CLP + GIK group exhibited decreased epithelial permeability of the ileum and increased expression of zonula occludens-1, occludin, and claudin-1 in the ileum. The findings demonstrated that the UCP2 and NLR family-pyrin domain-containing 3/caspase 1/interleukin 1*β* signaling pathway may be involved in intestinal barrier injury and that GIK treatment decreased intestinal barrier permeability. Thus, GIK may be a useful treatment for intestinal barrier injury during sepsis.

## 1. Introduction

Sepsis is a type of systemic inflammatory response syndrome that is caused by infection and is associated with a complex pathogenesis, high rate of mortality, and huge financial costs [1]. Although numerous efforts, such as the Surviving Sepsis Campaign, have been made in the past five decades, the mortality rate of sepsis is still the highest among critically ill patients. In United States, the morbidity of sepsis or septic shock is 750,000 cases per year, with a mortality rate of 20–40% [2].

The cases of intraperitoneal infection, such as appendicitis perforation, intestinal fistula, or necrotizing enterocolitis, take up to 20% of those of sepsis [3]. However the mortality of this kind of diseases nearly reaches 60% [3]. With regard to the development of sepsis, the intestine is special in that it is always the first organ to be involved and it may promote multiple organ dysfunction (MODF) [4]. The intestinal barrier, which is a physical barrier formed of various types of intestinal epithelial cells and intercellular tight junction complexes, may play a key role in MODF [4, 5]. At least three transmembrane proteins are required for the assembly of the tight junction complex, including occludin, claudin-1, and zonula occludens-1 (ZO-1). ZO-1 is essential for the formation of tight junctions due to its ability to interact with the other two tight junction complex proteins to stabilize the epithelial barrier [6, 7]. The production of cytokines has been proven to interfere with epithelial tight junction integrity and damage intestinal barrier function [6]. Therefore, toxic luminal antigens may leak into tissues and microorganisms in the gut may translocate through paracellular pathways into the circulation, and this may result in MODF [8].

Glucose-insulin-potassium (GIK) therapy has been reported to decrease mortality among critically ill patients due to its suppression of inflammatory and apoptotic effects and has been used clinically for decades [9]. Insulin, which has now been confirmed to be the major bioactive component of GIK, can decrease pyruvate production and glycolysis and increase glycogen synthesis, which may result in reduced production of lactate during sepsis [10]. Furthermore, insulin influences lipid metabolism and decreases lipid peroxidation during sepsis [11].

Uncoupling protein 2 (UCP2), a mitochondrial inner membrane protein, is widely expressed in various tissues, including intestinal tissue, and has been reported to have an important role in the maintenance of the intestinal mucosal barrier during inflammatory or ischemia/reperfusion conditions [12–14]. In isolated mitochondria, superoxides can activate UCP1, UCP2, and UCP3, and UCPs may in turn reduce the production of superoxide [15, 16]. However, this may be different under septic conditions. It is reported that the expression of UCP2 is elevated during sepsis and is adjusted by acetyl-CoA and pyruvate; the latter is the intermediate product of glucose metabolism and can produce lactate [17]. Recently, Rajanbabu reported that genipin, a selective inhibitor of UCP2, decreases the expression of the NLR family-pyrin domain-containing 3 (NLRP3) inflammasome via downregulation of UCP2 [18]. Additionally, Moon et al. demonstrated that the mortality rate of wild-type rats with cecal ligation and puncture- (CLP-) induced sepsis was higher than that of UCP2-knockout rats and that UCP2 may increase the expression of fatty acid synthase (FASN) and promote NLRP3 inflammasome activation during sepsis [19]. The findings of these studies imply that UCP2 may have adverse effects on survival during sepsis.

It has not been confirmed whether UCP2 plays a role in intestinal mucosal injury during septic conditions. However, there appears to be some relationship between insulin and UCP2. Therefore, the purpose of the present study was to confirm the potency of the effects of GIK using a rat model of intestinal mucosal barrier injury and to elucidate the association between GIK therapy and the expression of UCP2 in a CLP-induced sepsis model.

## 2. Materials and Methods

### 2.1. Animal Experiments

Male Sprague-Dawley (SD) rats (250–300 g) were obtained from the Experimental Animal Center of Southern Medical University (Guangdong, China). The Ethical and Research Committee of Southern Medical University approved the study. The experiments were carried out based on the Guide for the Care and Use of Laboratory Animals of the National Institutes of Health (NIH). The rats were randomly allocated into one of five groups (*n* = 6 in each group) as follows: control group, CLP group, sham surgery group, CLP + GIK group, and CLP plus glucose and potassium (GK) group. Jugular vein catheters were inserted in rats of the latter four groups one day before the surgery. Two sets of experimental models were conducted: in the first set, the samples were harvested and assessed 24 h following CLP, while the second set was used for mortality evaluation after 7 days.

The CLP procedure was conducted as described previously [20]. Briefly, rats were given free access to water but were deprived of food for 12 h prior to surgery. The rats were anaesthetized with chloral hydrate (0.35 mg/kg, intraperitoneal) and their abdominal hair was removed. Ten minutes later, skin disinfection and ventral midline incision (3 cm) were performed.

In the first set of rats (in which samples were harvested and assessed at 24 h), the following steps were carried out for the CLP groups in sequence: locating the cecum; ligating 25% of the distance from distal side of the cecum to the ileocecal valve, avoiding damage to the ileum and mesenteric vessels; puncturing the cecum through and through once using a 22-gauge needle; squeezing a fecal droplet; replacing the cecum; closing the incision; and injecting normal saline (5 ml/100 g) subcutaneously. The sham surgery group underwent the same procedure excluding the cecal ligation and puncture. GIK solution consisted of glucose (260 g/l), insulin (60 U/l), and potassium (60 mmol/l), and insulin was diluted with sterile water. GK solution contained the same components as GIK but without insulin and served as the base control therapy to test the effect of insulin. Immediately following the surgery, CLP + GIK group were injected with the GIK solution intravenously at a speed of 4 ml/kg/h for 24 h, while the CLP + GK group were given the GK solution at the same speed for 24 h. Afterward, the rats were placed in cages at room temperature (25°C) and given free access to food and water. The peripheral blood glucose levels of surviving rats were recorded every 3 h. The first set of rats was sacrificed at 24 h after the CLP procedure and the blood and ileum tissue specimens were harvested. Serum samples were collected and prepared for further assessment after blood specimen centrifugation at 6,000 ×g for 5 min at 4°C.

The second set of rats underwent the same CLP procedure as the first set, except for a ligation of 50% of the distance from the cecal distal side to ileocecal valve. There are 10 rats in control group or sham CLP group and 15 in the other three groups, respectively. In addition, the second set of rats that were treated with GIK or GK for 24 h was observed for 7 days, and the number of animals that died during this time was recorded.

### 2.2. Ileum Histological Analysis

Ileum tissues were fixed in 4% paraformaldehyde, paraffin embedded, sectioned at a thickness of 5 *μ*m, and subjected to hematoxylin and eosin (H&E) staining. Chiu's scoring system was applied to evaluate the extent of intestinal mucosal injury [21], which was graded from 0 to 5 as follows: grade 0: normal mucosal villi; grade 1: space in subepithelium with capillary hyperemia; grade 2: epithelium separating from mucosal lamina; grade 3: massive epithelial detachment from villi; grade 4: mucosal villi detachment with exposure of mucosal lamina; and grade 5: digestion of mucosal lamina with ulceration or bleeding.

### 2.3. Cytokine Measurement in Ileum Tissues and Sera

Ileum tissues (50 mg) were homogenized in 500 *µ*l sodium phosphate buffer (pH 7.4). After centrifugation at 6,000 ×g for 20 min at 4°C, the supernatants were extracted with acetone and dried. The supernatants were then resuspended in PBS for measurement. Subsequently, the following cytokines were measured by double-antibody ELISA Kits (R&D Systems, Minneapolis, MN, USA): interleukin (IL)-1*β*, IL-6, IL-10, and tumor necrosis factor-*α* (TNF-*α*). Serum samples were also processed in the same way as ileum supernatants except that only IL-1*β* and TNF-*α* were examined, in order to study the systemic proinflammatory cytokine levels. The results for all specimens were measured using spectrophotometer at 450 nm in 96-well microplates.

### 2.4. Intestine Permeability Testing

FITC-labeled dextran (FD4; Sigma-Aldrich, USA) was used to assess intestinal permeability, as described previously [22]. FD4 (1 ml; 5 mg/ml) was administered into a 10 cm long section of ileum following ligation at both sides, and 4 ml serum was collected 1 h later. Standard curves were produced using serial dilutions of FD4, and the actual serum concentrations (expressed as *µ*g/ml) were calculated based on the levels of fluorescence determined by a multifunctional microplate reader (SpectraMax M5; Molecular Devices) with excitation at 490 nm and emission at 530 nm.

### 2.5. Western Blot Analysis

Total protein from ileum tissues (30 mg) was extracted using a Total Protein Extraction Kit (Keygen Biotech, China) and the protein concentration from the supernatants was measured by BCA Protein Assay (Keygen Biotech). Total protein samples (50 *µ*g) were boiled in water for 5 min after mixing with 5x loading buffer and then measured by western blotting with polyclonal rabbit antioccludin (dilution, 1 : 1,000; Abcam, Cambridge, MA, USA), polyclonal rabbit anti-ZO-1 (dilution, 1 : 250; Abcam), polyclonal rabbit anticlaudin-1 (dilution, 1 : 500; Abcam), polyclonal rabbit anti-UCP2 (dilution, 1 : 500; Santa Cruz Biotechnology, Inc., Santa Cruz, CA, USA), polyclonal rabbit anti-NLRP3 (dilution, 1 : 500; Abcam), monoclonal rabbit anticaspase-1 pro/p10/p12 (dilution, 1 : 1,000; Abcam), polyclonal rabbit anti-ASC (dilution, 1 : 1,000, Santa Cruz), and polyclonal rabbit anti-*β*-actin (dilution, 1 : 2,000; ProteinTech) antibodies. Peroxidase-conjugated goat anti-rabbit IgG (dilution, 1 : 2,000; ProteinTech) was used as the secondary antibody. Following incubation with the primary and secondary antibodies, the PVDF membranes (EMD Millipore, USA), were washed three times with TBST and scanned using an Imaging System (Kodak, Japan). Densitometric analysis was performed with ImageJ software (NIH).

### 2.6. Statistical Analysis

All data were acquired from at least 6 independent experiments and are presented as the mean ± standard deviation unless otherwise indicated. Statistical significance was determined by unpaired *t*-test for comparisons between two groups, or one-way ANOVA followed by Bonferroni's multiple comparisons test for multiple groups. The *χ*^2^ test was adopted for comparison of categorical variables. Surviving rate was illustrated with Kaplan-Meier curves and tested using the log rank method. The number of samples is shown in the figures. GraphPad Prism 5.0 Software was used for statistical analysis and *P* < 0.05 was considered to indicate statistical significance.

## 3. Results

### 3.1. GIK Therapy Lowers Mortality in Rats Subjected to CLP-Induced Sepsis

**Figure 1:**
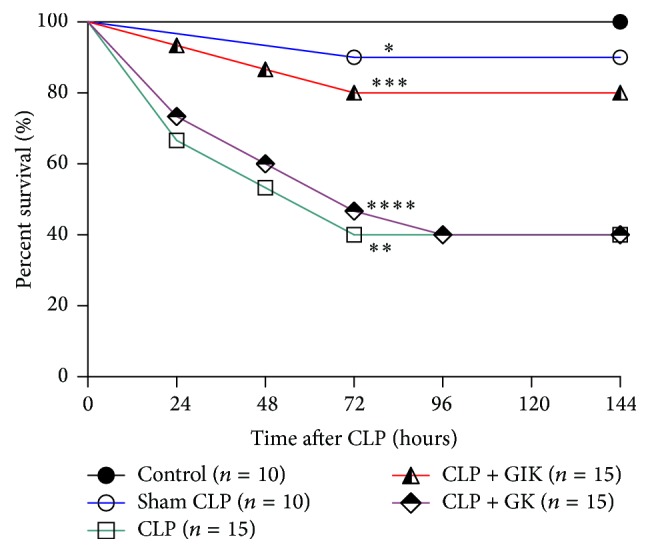
Effect of GIK on surviving rate of each group in second set of rats 7 days following CLP procedure. All the values are from no less than six independent experiments. Most deaths in each group happened during the first 3 days and CLP disposal led to the highest mortality of up to 60% during 7-day period. ^*∗*^*P* > 0.05 versus control group, ^*∗∗*^*P* < 0.05 versus. sham CLP group, ^*∗∗∗*^*P* < 0.05 versus CLP group, and ^*∗∗∗∗*^*p* > 0.05 versus CLP group.

To investigate the effect of GIK on mortality following CLP-induced sepsis, Kaplan-Meier curves were produced for the 7 days following the CLP procedures. As illustrated in Figure 1, most of the mortalities in each group occurred during the first 3 days and CLP led to the highest mortality rate of up to 60% during the 7-day period (*P* < 0.01 versus control group). After GIK therapy, the mortality rate of rats during sepsis was reduced to ~20%, which was significantly lower than that in the CLP group (*P* < 0.05). However, GK therapy did not achieve such an effect (*P* < 0.05 versus CLP + GIK group).

### 3.2. GIK Therapy Lowers Serum IL-1*β* and TNF-*α* Production following CLP-Induced Sepsis

**Figure 2:**
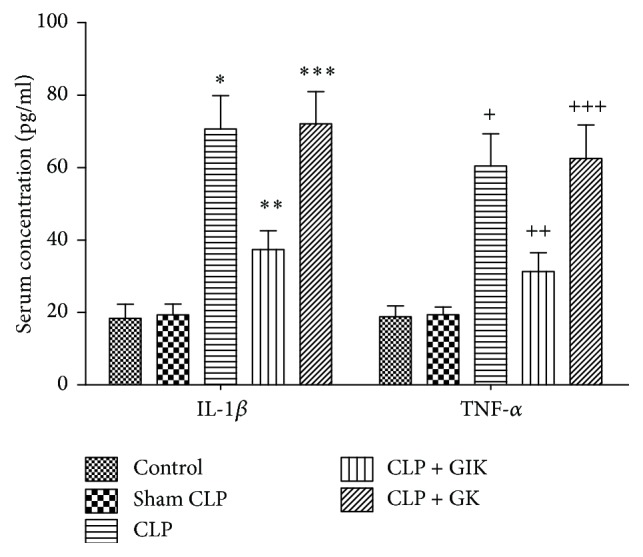
Effects of GIK on serum IL-1*β* and TNF-*α*cytokines production in CLP-induced polymicrobial sepsis rats. Sera were harvested 24 hours after CLP challenge in first set of rats. All the values are expressed as mean ± SD (*n* = 6 in each analysis) of no less than six independent experiments. IL-1*β*: ^*∗*^*P* < 0.01 versus sham CLP group, ^*∗∗*^*P* < 0.01 versus CLP group, ^*∗∗∗*^*P* > 0.05 versus CLP group; TNF-*α*: ^+^*P* < 0.01 versus sham CLP group, ^++^*P* < 0.01 versus CLP group, and ^+++^*P* > 0.05 versus CLP group.

Sepsis is caused by infection and is marked by the elevation of various serum cytokine levels. As shown in Figure 2, proinflammatory cytokines, including IL-1*β* and TNF-*α*, were dramatically elevated in the CLP group compared with the control or sham CLP group (both *P* < 0.01). GIK therapy, but not GK therapy, attenuated these effects (*P* < 0.01 versus CLP group or CLP + GK group, resp.).

### 3.3. GIK Therapy Decreases Intestinal Mucosal Injuries following CLP-Induced Sepsis

**Figure 3:**
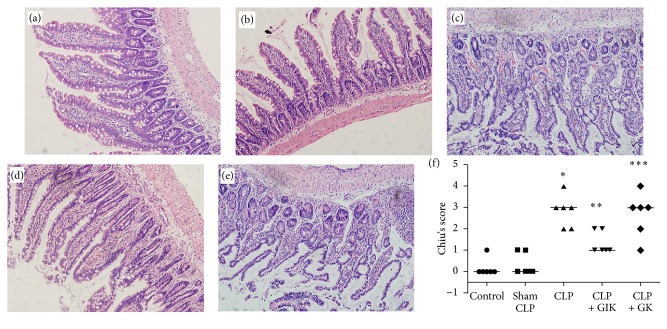
Effects of GIK on histopathological changes in ileum tissues in CLP-induced polymicrobial rats. Ileum tissues were carried out for histological evaluation at 24 hours after CLP in first set of rats. Representative H&E staining histological changes of ileum harvested from rats of different groups. (a) Control group, (b) sham CLP group, (c) CLP group, (d) CLP + GIK group, and (e) CLP + GK group (hematoxylin and eosin staining, magnification 40x). (f) shows Chiu's scoring of mucosal injury of each experimental group (*n* = 6 in each analysis) of no less than six independent experiments (^*∗*^*P* < 0.01 versus sham CLP group, ^*∗∗*^*P* < 0.01 versus CLP group, and ^*∗∗∗*^*P* < 0.01 versus CLP + GIK group by the chi-square test for trend).

As shown in Figure 3, scoring of H&E-stained ileum tissues using Chiu's method revealed that there was a sharp rise in intestinal mucosal injury in the CLP group at 24 h after the CLP procedure compared with the control or sham surgery groups (both *P* < 0.05). GIK therapy significantly alleviated mucosal damage compared to the CLP group (*P* < 0.05), while GK therapy did not have the same effect as GIK therapy (*P* < 0.05).

### 3.4. GIK Therapy Lowers Proinflammatory Cytokine Production and Increases Anti-Inflammatory Cytokine Production following CLP-Induced Sepsis

**Figure 4:**
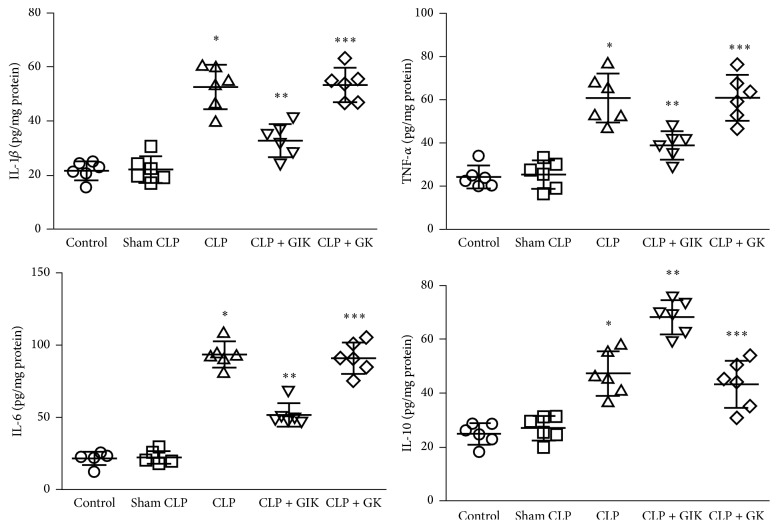
Cytokines production of intestinal tissues, including IL-1*β*, IL-6, TNF-*α*, and IL-10, was influenced by GIK in CLP-induced polymicrobial sepsis rats. Ileum tissues were harvested 24 hours after CLP challenge in first set of rats. All the values are expressed as mean ± SD (*n* = 6 in each analysis) of no less than six independent experiments. ^*∗*^*P* < 0.01 versus sham CLP group, ^*∗∗*^*P* < 0.01 versus CLP group, and ^*∗∗∗*^*P* < 0.01 versus CLP + GIK group.

The intestinal mucosal immune system is extensive in the body, secondary only to skin tissues, and comprises large amounts of inflammatory cells, including lymphocytes, monocytes, and macrophages. These inflammatory cells produce high quantities of pro- or anti-inflammatory cytokines, including IL-1*β*, IL-6, TNF-*α*, and IL-10. In the present study, all of these cytokines were used to assess the level of intestinal inflammation (Figure 4). At 24 h after CLP, a significant increase in the production of pro- and anti-inflammatory cytokines in ileum tissue was detected in the CLP group compared with the control group or sham CLP group (*P* < 0.01). GK therapy could not attenuate this adverse effect. However, GIK treatment was shown to significantly decrease proinflammatory cytokine levels, including IL-1*β*, IL-6, and TNF-*α*, and increase IL-10 production, compared with the levels in the CLP or CLP + GK group (both *P* < 0.01).

### 3.5. GIK Therapy Decreases Intestine Permeability following CLP-Induced Sepsis

**Figure 5:**
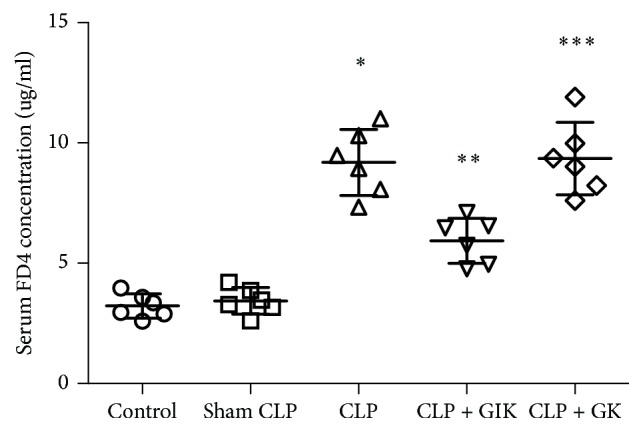
Serum FD4 concentration was influenced by GIK in CLP-induced polymicrobial sepsis rats. GIK decreases intestinal epithelial permeability obviously. Sera were collected 24 hours after CLP challenge in first set of rats. All the values are expressed as mean ± SD (*n* = 6 in each analysis) of at least six independent experiments. ^*∗*^*P* < 0.01 versus sham CLP group, ^*∗∗*^*P* < 0.01 versus CLP group, and ^*∗∗∗*^*P* < 0.01 versus CLP + GIK group.

Serum concentrations of FD4 were used to assess the degree of intestinal permeability; the higher the serum concentration of FD4, the greater the intestinal permeability. As can be seen from Figure 5, the FD4 level was significantly elevated in the CLP group compared with the sham or control groups at 24 h following CLP (*P* < 0.05), and this was not reduced by GK (*P* < 0.01). However, after GIK therapy, the FD4 level decreased dramatically (*P* < 0.01).

### 3.6. GIK Therapy Increases the Expression of ZO-1, Occludin, and Claudin-1 Protein following CLP-Induced Sepsis

**Figure 6:**
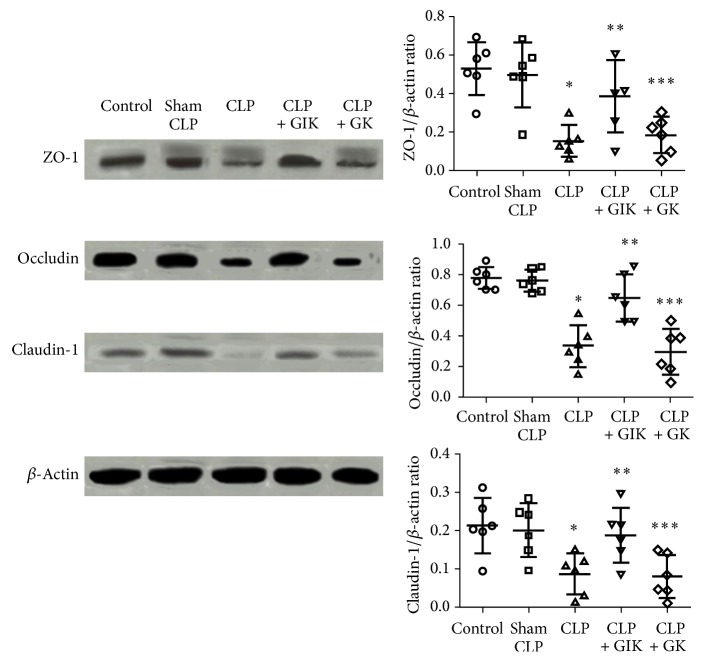
Expression of intestinal tight junction proteins was influenced by GIK in CLP-induced polymicrobial sepsis rats. Ileum tissues were harvested 24 hours after CLP challenge in first set of rats. All the values are expressed as mean ± SD (*n* = 6 in each analysis) of no less than six independent experiments. ^*∗*^*P* < 0.01 versus sham CLP group, ^*∗∗*^*P* < 0.01 versus CLP group, and ^*∗∗∗*^*P* < 0.01 versus CLP + GIK group.

ZO-1, occludin, and claudin-1 proteins are components of intestinal epithelial tight junctions. Their expression is always decreased by injuries such as sepsis, burn, and bleeding, and this may lead to intestinal mucosal barrier injuries [6].  Figure 6 shows that there was a sharp decline in the expression levels of all three proteins in the CLP and CLP + GK groups compared with the control or sham group (all *P* < 0.01); however, GIK treatment increased these expression levels significantly compared with the CLP group (*P* < 0.01).

### 3.7. GIK Therapy Decreases the Expression of UCP2, NLRP3, and Caspase-1 p10 Protein following CLP-Induced Sepsis

**Figure 7:**
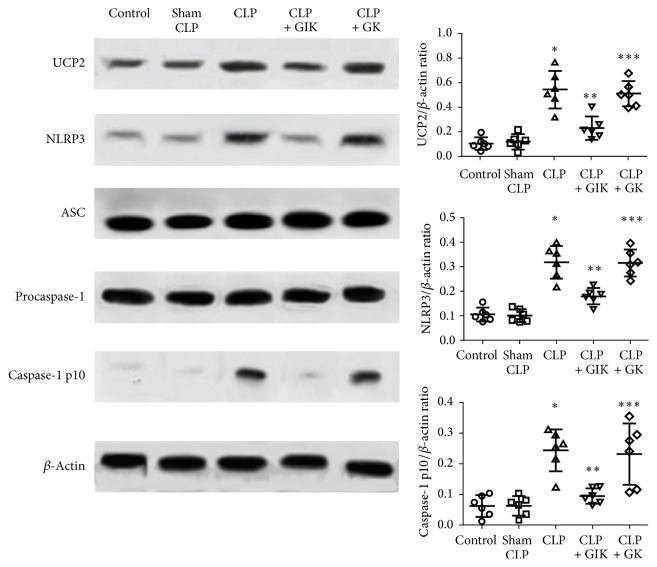
GIK inhibits CLP-induced UCP2 expression and NLRP3 and caspase-1 p10 activation. All the values are expressed as mean ± SD (*n* = 6 in each analysis) of no less than six independent experiments in first set of rats. ^*∗*^*P* < 0.01 versus sham CLP group, ^*∗∗*^*P* < 0.01 versus CLP group, and ^*∗∗∗*^*P* < 0.01 versus CLP + GIK group.

The association between UCP2 and NLRP3/caspase-1/IL-1*β* signaling pathway was clarified recently, and it was shown that upregulating UCP2 expression can induce proinflammatory effects by activating the NLRP3/caspase-1/IL-1*β* signaling pathway [19]. To investigate the anti-inflammatory mechanism of GIK and its relationship with UCP2, UCP2 and NLRP3/caspase-1/IL-1*β* signaling pathway were investigated. As demonstrated in Figure 7, CLP induced a significant increase in the expression levels of UCP2, NLRP3, and caspase-1 p10 protein (*P* < 0.01). However, GIK treatment significantly downregulated the CLP-induced increase in UCP2 expression and suppressed NLRP3 and caspase-1 p10 activation (*P* < 0.01).

## 4. Discussion

Sepsis is still associated with a high incidence and mortality due to its complex pathophysiology and lack of sufficient therapy [23]. Injury of the intestinal mucosal barrier is considered to have a key role in the occurrence and development of sepsis and septic shock [24]. CLP, which mimics perforated appendicitis in humans leading to peritonitis and sepsis, is a common model of sepsis that is able to produce intestinal mucosal barrier injury [22, 25]. The present study demonstrates that GIK protects intestinal mucosal barrier function in CLP-induced sepsis and that UCP2 and the NLRP3/caspase-1/IL-1*β* signaling pathway may be involved in this process.

GIK, an effective prescription for coronary heart disease and other heart diseases clinically [9, 26, 27], has been shown to exert anti-inflammatory effects to attenuate the systemic inflammatory response in endotoxemia rats and humans by inactivating nuclear factor *κ*B or the phosphoinositide 3-kinase/Akt signaling pathway [28]. GIK is a “cocktail” recipe that consists of dextrose, insulin, and potassium: insulin has a bioactive role in the anti-inflammatory, antioxidation, and antiapoptotic effects in various conditions; dextrose resists hypoglycemia; insulin and dextrose together assist the transfer of potassium from extracellular to intracellular fluids; and all three components work together to exert bioactive effects without the side effects of hypoglycemia and hypokalemia [29]. Our study is the first to demonstrate that GIK decreased the rate of mortality in a rat model of sepsis, decreased the production of intestinal tissue proinflammatory cytokines, including IL-1*β*, IL-6 and TNF-*α*, and increased the level of IL-10. In addition, GIK increased the expression of tight junction proteins, including ZO-1, occludin, and claudin-1, during sepsis and thus decreased the intestinal permeability. Furthermore, histological analysis also showed that GIK attenuates the intestinal epithelial mucosal injury when suffering sepsis. Finally, GIK may work by downregulating the expression of UCP2 and inactivating the NLRP3/caspase-1/IL-1*β* signaling pathway.

UCP2 and NLRP3 are widely expressed in the intestines and may be activated by various types of stimuli [30]. UCP2, a mitochondrial inner membrane protein, is capable of regulating cell metabolism of carbohydrates and lipids that have central roles in sepsis or ischemia/reperfusion diseases [31]. In the past, UCP2 was considered to be a protective factor providing resistance to sepsis or ischemia/reperfusion diseases [32, 33]. However, recent studies show that UCP2 can increase the susceptibility of rats to lipopolysaccharides during septic conditions [34, 35] and that UCP2 could upregulate the expression of FASN and increase the production of triglycerides, ultimately activating the NLRP3/caspase-1/IL-1*β* signaling pathway leading to an increased rate of mortality in rats [19]. UCP2 may be upregulated by high glucose stress and glucose and lipid metabolites, which are always elevated during sepsis [36, 37]. Additionally, the expression of UCP2 can be directly elevated by pyruvate [37], the intermediate product of lactate, which is strongly associated with sepsis. Insulin, the key component of GIK, is also closely related to the metabolism of carbohydrates and lipids and is thought to decrease production of pyruvate, high glucose stress, and lipid peroxidation during septic conditions. All of these may explain why UCP2 is involved in the anti-inflammatory effects of GIK.

The intestinal mucosal barrier mainly consists of epithelial cells and intracellular junctions, of which tight junctions play the most important role. Tight junctions are composed of tight junction proteins, including at least occludins, claudins, and ZO-1 [38]. The present study demonstrates that, under septic conditions, proinflammatory cytokines may reduce the expression of tight junction proteins, induce the apoptosis of epithelial cells, and, thus, lead to an increase in the permeability of the intestinal mucosal barrier. UCP2 can be upregulated by pyruvate, which is an intermediate product of lactate, and our study indicates that this leads to the activation of the NLRP3 inflammasome, resulting in damage to the intestinal mucosal barrier. GIK therapy decreases the expression of UCP2 and the NLRP3 inflammasome.

In conclusion, this study demonstrated that GIK alleviates intestinal mucosal barrier injury during CLP-induced sepsis for the first time. UCP2 and the NLRP3/caspase-1/IL-1*β* signaling pathway may be involved in this process. These results imply that GIK may be a useful treatment for intestinal mucosal barrier injury.
